# Limberg Transpositional Fasciocutaneous Flap in Sacrococcygeal Pilonidal Sinus Disease (SPSD): A Case Series

**DOI:** 10.7759/cureus.61086

**Published:** 2024-05-25

**Authors:** Tushar Dahmiwal, Anup Zade, Darshana Tote, Srinivasa Reddy, Nikhil Thatipalli, Jhanwi Khurana, Kesav Sudabattula, Shailabh Bhadra

**Affiliations:** 1 Surgery, Jawaharlal Nehru Medical College, Datta Meghe Institute of Higher Education and Research, Wardha, IND

**Keywords:** sacrococcygeal pilonidal sinus disease, pilonidal sinus, serous discharge, rhomboid flap, limberg flap

## Abstract

Introduction

Among young male adults, sacrococcygeal pilonidal sinus disease (SPSD) is a prevalent condition. There are several possibilities for treatment, including both conservative and surgical methods. Medical supervision or conservative management is not the cutting-edge and preferred management nowadays. Although not fatal, it negatively impacts young people's quality of life in terms of schooling and means of subsistence and is socially awkward.

Method

About 10 individuals in this case series have serous drainage from the sinus in the sacral region, which is a common symptom. The patients were entitled to a full recovery from their illness. In all these patients, the Limberg flap procedure was recommended, and just one patient out of 10 had a minimal infection. Every patient was satisfied with how the surgery turned out. Overall, the Limberg flap (rhomboid flap) approach is becoming the norm for care since it has a lower rate of recurrence, fewer postoperative problems, and a shorter learning curve.

Result

Flap necrosis instances were absent in all the cases. And out of 10 cases, one patient came with a surgical site infection during the follow-up, suggesting a complication rate of 10%.

Conclusion

For the treatment of primary pilonidal illness, rhomboid excision utilising the Limberg transpositional fasciocutaneous flap technique is seen as a safer option that encompasses numerous sinuses. It requires less time in the hospital and has fewer postoperative problems.

## Introduction

The Latin term for pilonidal sinus is "nest of hairs." It usually affects young adults and masculine, hirsute individuals. The United States of America (USA) has a frequency of 26/100,000, while Asia has a prevalence of approximately 6.6% [1]. Over time, the understanding of the pathogenesis of the disease has changed from a congenital to a conclusive acquired aetiology. High hair volume, intense force, and susceptibility to infection were the three main contributing variables [2]. After secondary infection, many subcutaneous sinuses and abscesses develop. The primary difficulty at hand is the disease's recurrence. The Limberg approach, which basically consists of a flap treatment that obliterates the natal cleft and accomplishes an off-midline closure, is used to prevent recurrence. Other common surgical therapeutic approaches include sinus tract excision with closure or healing by secondary intention [2,3].

## Materials and methods

Ten patients with primary pilonidal sinus, with a median follow-up duration of 11 months, were enrolled in the study after receiving consent and underwent Limberg flap procedures in our hospital between December 2022 and December 2023. The patients' mean age was 23.6 years. The BMI of all the patients were between 18.5 and 24.9 (normal) except for one patient who had a BMI of 32. A retrospective observational study was done during follow-ups.

Only clinical diagnoses were made for each patient. All patients included had purulent sinus discharge from the sacrococcygeal region, which was associated with pain that persisted for about five to 10 days when they first presented with the symptoms. But the illness progressed in a waxing and waning manner over about a year and a half. Upon visual inspection, solitary tract sinus apertures were found in the midline natal cleft. In all the cases, the primary sinus orifice was situated between 4 and 7 cm in the midline and restricted in the navicular area (Type 3 as per the Tezel classification of pilonidal sinus disease) (Table 1).

**Table 1:** Tezel classification

Type	Tezel classification
Type 1	Asymptomatic sinus
Type 2	Acute pilonidal abscess
Type 3	Chronic (symptomatic) restricted in the navicular area
Type 4	Chronic (symptomatic) exceeded in the navicular area
Type 5	Recurrent pilonidal sinus

Preoperative intravenous ceftriaxone (1 gram) was given in accordance with standard surgical protocol. The patient was given spinal anaesthesia and put in the jack-knife position. Adhesive tapes were used to retract the buttocks so the surgical site could be seen. 10% Betadine was used to sterilise the operative site. The sinus tract was injected with methylene blue dye. All of the central sinus orifices were included in the rhomboid excision procedure.

**Figure 1:**
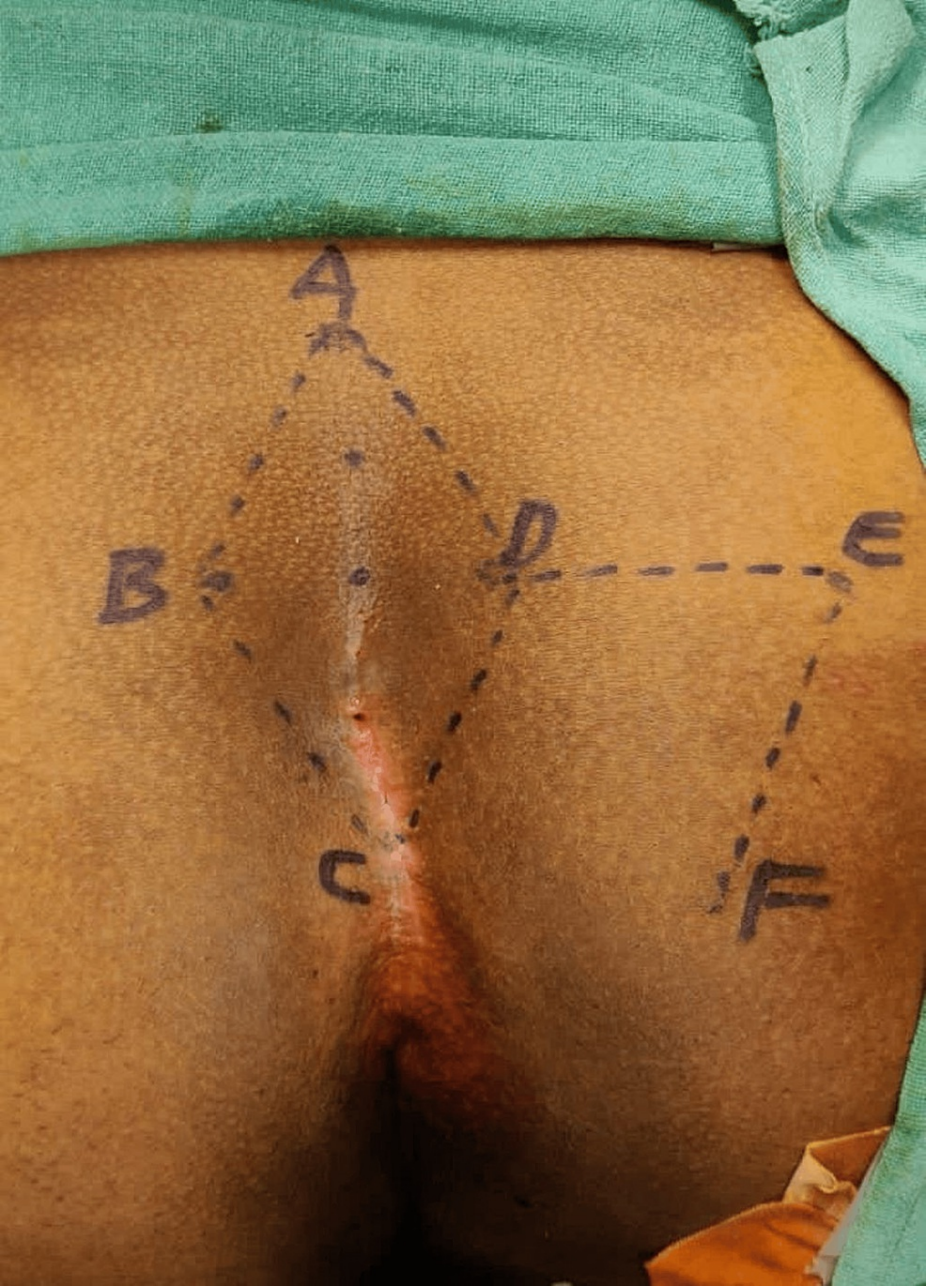
Incision site

As per the Limberg flap concept, a set of communicating equilateral triangles makes up the Limberg flap. Since every angle is 60°, the length of every side of the defect and the flap is the same. A flap of the same size as the defect that needs to be eliminated is created by this orientation. The rhomboid's long axis in the midline was designated with the letters A through C, with C next to the perianal skin and A positioned to allow for the removal of all concerning tissues. At 60% of its length, the line BD makes a right angle crossing across the midpoint of AC. DE was the same length and was a straight extension of line BD. EF had the same length and ran parallel to DC, thus marking the incision site (Figure 1).

For instance, if AC = 10 cm, BD transects at 6 cm of AC. The lines BC and DC were marked convexly and concavely, respectively, to lateralize the flap's bottom end (C) and avoid the inter-gluteal cleft.

**Figure 2:**
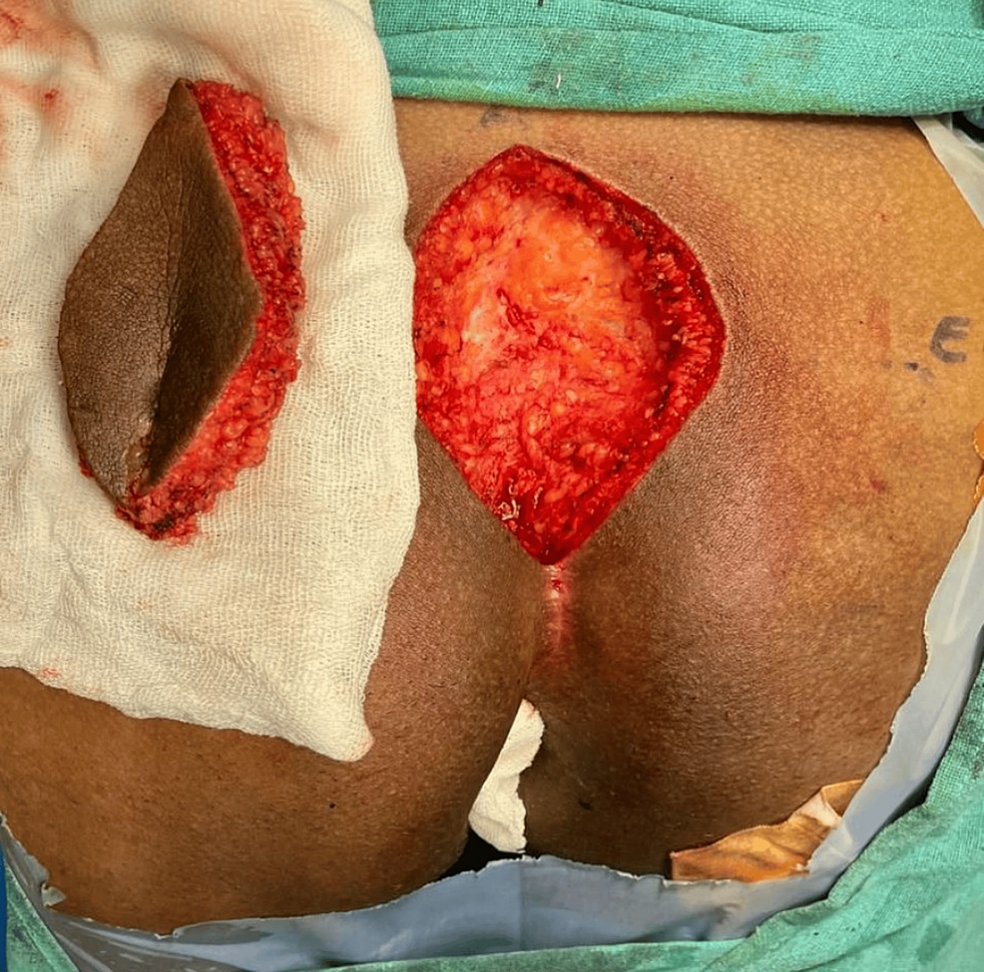
Rhomboid excision up to the pre-sacral fascia

Mono-polar diathermy cautery was used to dissect the rhomboid excision up to the pre-sacral fascia (Figure 2).

**Figure 3:**
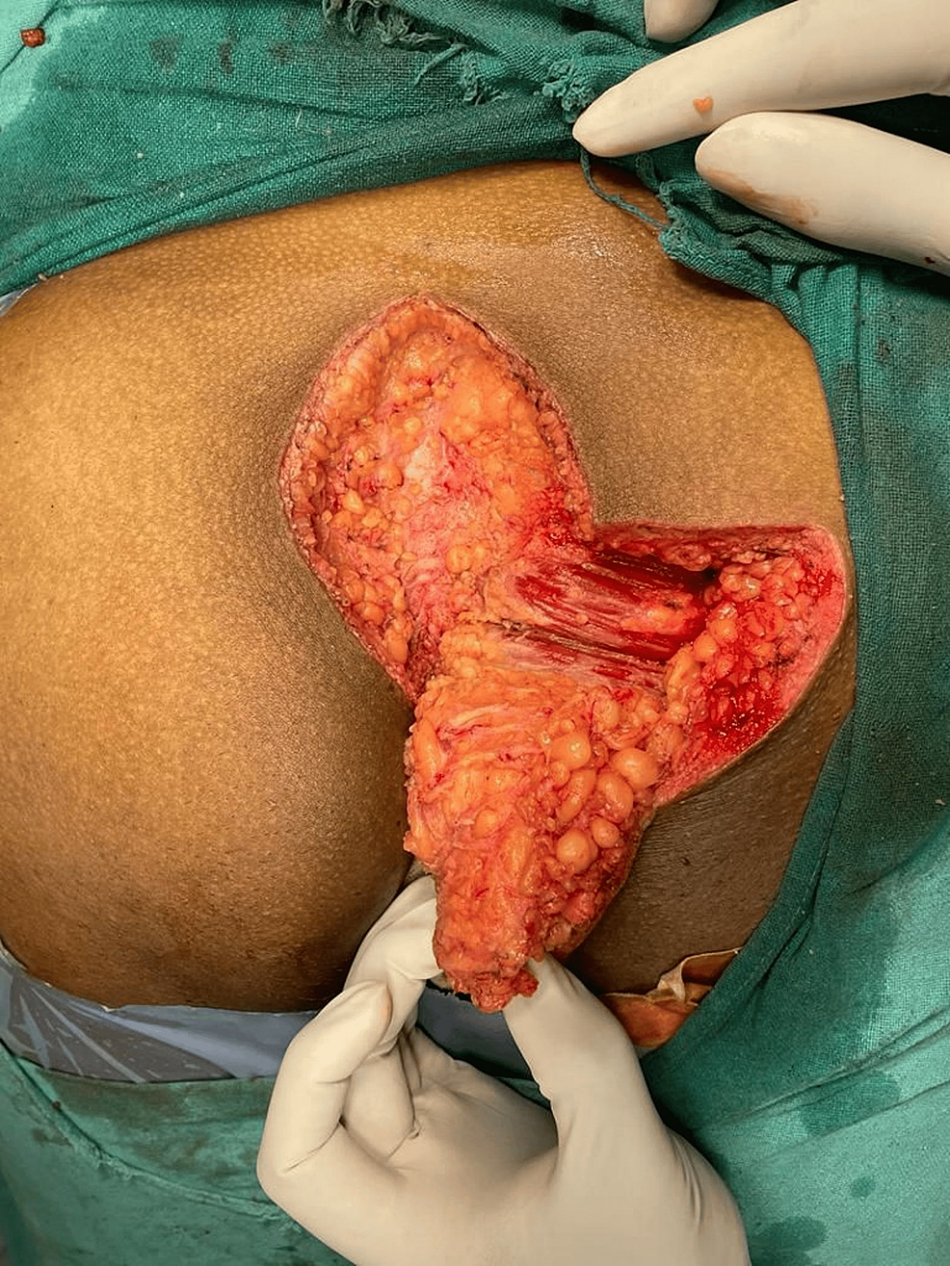
Transpositional flaps raised

The skin, subcutaneous tissue, and gluteal fascia were all fashioned into the transpositional flaps DE and EF (Figure 3).

**Figure 4:**
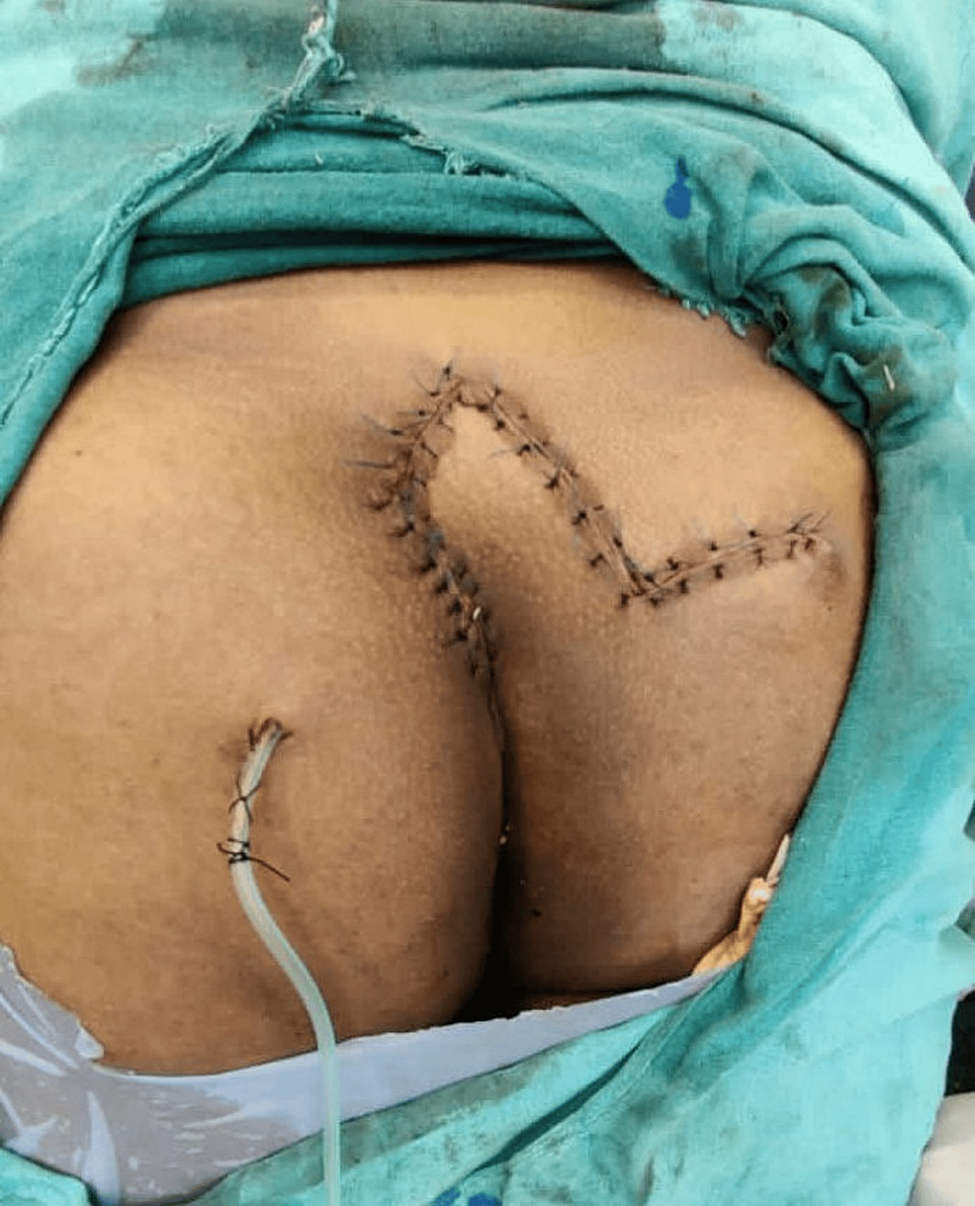
Final closure of the Limberg transpositional fasciocutaneous flap with a negative suction drain in situ

To prevent flap necrosis, the vascularity of the flap edges was reevaluated. Following the installation of a negative suction drain, the subcutaneous tissue was closed using Vicryl 2-0. Ethilon 2-0 was used for skin closure (Figure 4).

There was no tension in the flap closure.

**Figure 5:**
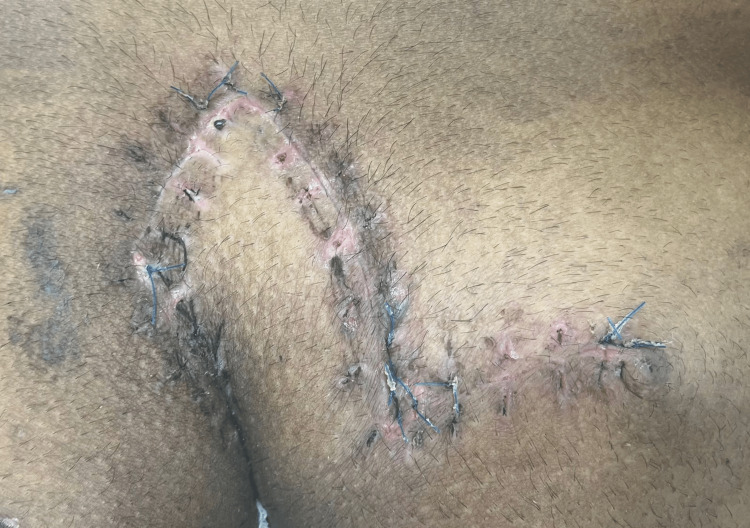
Alternate suture removal of the operated site

During the postoperative phase, all the patients were nursed while lying prone up to postoperative day (POD)-4. On POD-5, a negative suction drain was removed after the discharge was <10 ml per day. Alternate suture removal was done on POD-13 (Figure 5).

**Figure 6:**
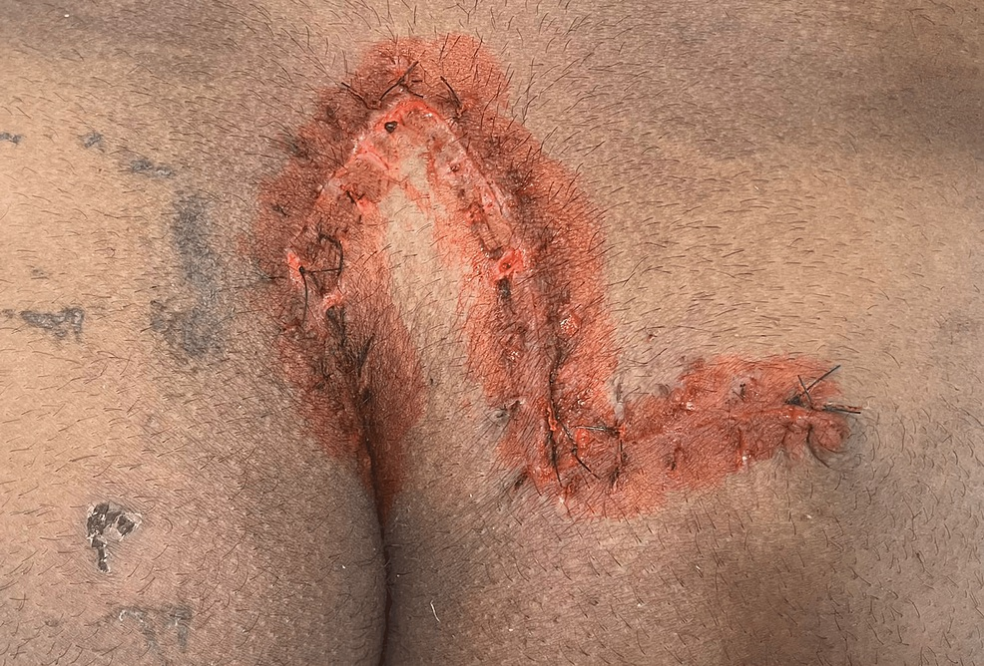
Complete suture removal of the operated site

It was followed by complete suture removal (Figure 6).

In other cases also, the drain was removed after the discharge was less than 10 ml, and sutures were removed accordingly on POD-14/POD-15 as built was different for different patients. Hence, depending upon the drain output (<10 ml), the negative suction drain was removed, and depending upon the suture line, suture removal was done. 

Patients were also counselled about the slight lifestyle modifications as per their occupation and working pattern.

## Results

Flap necrosis instances were absent in all the cases. And out of 10 cases, one patient came with a minimal surgical site infection (minimal) during the follow-up, suggesting a complication rate of 10% (Table 2).

**Table 2:** Summary of all the cases M: male; F: female

Sr. no.	Case	Sex	Age (in years)	Follow-up	Postoperative stay	Wound infection
1	Case 1	M	18	11 months	5 days	Nil
2	Case 2	M	17	12 months	5 days	Nil
3	Case 3	M	24	13 months	6 days	Nil
4	Case 4	F	19	14 months	5 days	Nil
5	Case 5	F	21	12 months	7 days	Nil
6	Case 6	M	34	2 months	10 days	Present
7	Case 7	M	27	10 months	8 days	Nil
8	Case 8	M	28	9 months	9 days	Nil
9	Case 9	F	23	11 months	7 days	Nil
10	Case 10	M	25	10 months	6 days	Nil

Thus, to conclude, the Limberg transpositional fasciocutaneous flap is a great and optimal option with very little hospital stay and postoperative complications, along with reduced chances of recurrence.

## Discussion

There are various benefits to the Limberg or rhomboid flap restoration, which is what this case series has used. Dr. Alexander Limberg created the original concept for it in 1948. The stress created when the flap was moved to its new location was reduced by this design. Very less cases of recurrence (<5% in most trials), few problems, and strong patient/social acceptance are linked to the Limberg flap and its variations [4,5]. Only one patient out of 10 in a related trial by Jawade and Bande reported having a small wound infection, which is consistent with the results [6].

Comparing a case series of nine patients in another study by Jain and Thambuchetty, the complication rate was 15.79%, whereas the current case series has a complication rate of 10% [7].

Due to the possibility of a large excision region, it is appropriate for treating both numerous sinuses and recurrent illnesses. The location of the drain is another element that has a positive effect and speeds up the healing process [7]. In cases of pilonidal illness, general surgeons have various methods at their disposal. The treatment for acute pilonidal sinus illness involves draining the abscess and making an incision; this has a 15-40% recurrence rate [8]. Later on, these individuals need a second, conclusive procedure. Surgeons most commonly use a procedure called local excision without primary closure, which takes longer to heal since it has a secondary aim of healing the wound.

There are a one- to three-month recovery period and a postponed return to work. The range of recurrence rates is 2-35% [8,9]. Following extensive excision, the Karydakis flaps, the Bascom flap, the Limberg flap, and the V-Y advancement flap are among the reconstructive procedures used.

The Bascom cleft lift operation involves only the excision of midline pits and does not involve the removal of deep tissues [10]. It is a simpler treatment. Unlike the typical Limberg flap, which places the apices in the midline, the modified Limberg flap is an off-midline closure approach in which the cephalic and caudal apexes of the rhomboid are positioned 2 cm off the midline [11].

Regarding sacrococcygeal pilonidal sinus disease (SPSD), a number of surgical procedures have been documented; nevertheless, there hasn't been consensus regarding the best effective treatment because of the potential for complications and recurrences [8,12].

With fewer complications and recurrence rates, the modified Limberg flap procedure is thought to be a safer and more effective treatment for SPSD. When radical excision results in a significant defect, it is a very helpful procedure for complex sinuses with multiple pits and long corridors because it flattens the natal cleft with a wide, well-vascularized pedicle [9,11]. Nonetheless, a study found that the Karydakis flap performed better than the Limberg flap [8].

The Limberg flap is superior to open excision with secondary healing [12,13]. The shorter hospital stay in the current cases supports the notion that patients recovered rather well in the first week after surgery.

## Conclusions

For the treatment of primary pilonidal illness, rhomboid excision utilising the Limberg transpositional fasciocutaneous flap technique is seen as a safer option that encompasses numerous sinuses. It requires less time in the hospital and has fewer postoperative problems.
